# Functional analyses of heterotrimeric G protein G*α* and G*β* subunits in *Gibberella zeae*

**DOI:** 10.1099/mic.0.2007/012260-0

**Published:** 2008-02

**Authors:** Hye-Young Yu, Jeong-Ah Seo, Jung-Eun Kim, Kap-Hoon Han, Won-Bo Shim, Sung-Hwan Yun, Yin-Won Lee

**Affiliations:** 1 School of Agricultural Biotechnology and Center for Agricultural Biomaterials, Seoul National University, Seoul 151-921, Republic of Korea; 2 Department of Pharmaceutical Engineering, Woosuk University, Wanju 565-701, Republic of Korea; 3 Department of Plant Pathology and Microbiology, Texas A&M University, College Station, TX 77843-2132, USA; 4 Department of Medical Biotechnology, Soonchunhyang University, Asan 336-745, Republic of Korea

## Abstract

The homothallic ascomycete fungus *Gibberella zeae* (anamorph: *Fusarium graminearum*) is a major toxigenic plant pathogen that causes head blight disease on small-grain cereals. The fungus produces the mycotoxins deoxynivalenol (DON) and zearalenone (ZEA) in infected hosts, posing a threat to human and animal health. Despite its agricultural and toxicological importance, the molecular mechanisms underlying its growth, development and virulence remain largely unknown. To better understand such mechanisms, we studied the heterotrimeric G proteins of *G. zeae*, which are known to control crucial signalling pathways that regulate various cellular and developmental responses in fungi. Three putative G*α* subunits, *GzGPA1*, *GzGPA2* and *GzGPA3*, and one G*β* subunit, *GzGPB1*, were identified in the *F. graminearum* genome. Deletion of *GzGPA1*, a homologue of the *Aspergillus nidulans* G*α* gene *fadA*, resulted in female sterility and enhanced DON and ZEA production, suggesting that *GzGPA1* is required for normal sexual reproduction and repression of toxin biosynthesis. The production of DON and ZEA was also enhanced in the *GzGPB1* mutant, suggesting that both G*α* *GzGPA1* and G*β* *GzGPB1* negatively control mycotoxin production. Deletion of *GzGPA2*, which encodes a G*α* protein similar to *A*. *nidulans* GanB, caused reduced pathogenicity and increased chitin accumulation in the cell wall, implying that *GzGPA2* has multiple functions. Our study shows that *G. zeae* heterotrimeric G protein subunits can regulate vegetative growth, sexual development, toxin production and pathogenicity.

## INTRODUCTION

*Gibberella zeae* (anamorph: *Fusarium graminearum*) is an important fungal pathogen of small-grain cereal crops, such as barley, wheat and rice, and is distributed worldwide (Desjardins, 2006; Goswami & Kistler, 2004; Leslie & Summerell, 2006). This homothallic ascomycete reproduces both asexually and sexually, resulting in the production of macroconidia and ascospores, respectively, as major inoculum sources for plant infection (Goswami & Kistler, 2004). *G. zeae* produces a variety of toxic secondary metabolites, notably deoxynivalenol (DON) and zearalenone (ZEA), that threaten human and animal health (Desjardins, 2006). Diverse virulence factors such as mycotoxins, cyclic peptides, amino acids and a lipase have been shown to play crucial roles during pathogenesis in *G. zeae* (Han *et al.*, 2004; Kim *et al.*, 2007; Lu *et al.*, 2003; Oide *et al.*, 2006; Proctor *et al.*, 1995; Seong *et al.*, 2005; Voigt *et al*., 2005). However, the complex signalling mechanisms that regulate virulence in *G. zeae* are at best poorly understood. To develop innovative control strategies for *Fusarium* head blight, a better understanding is needed of the molecular mechanisms that underpin virulence gene regulation in *G. zeae*.

One conserved signalling pathway in filamentous fungi that needs further investigation in relation to *G. zeae* virulence is the heterotrimeric G protein-mediated signalling pathway. Heterotrimeric G protein-mediated signal perception and propagation are conserved from lower eukaryotes to humans (Dohlman & Thorner, 2001; Lengeler *et al.*, 2000). This signalling pathway plays a central role in controlling cell growth, development, virulence and secondary metabolite production in fungi (Bölker, 1998; Lengeler *et al.*, 2000). The basic units of this pathway are a G protein-coupled receptor, G proteins (consisting of G*α*, G*β* and G*γ* subunits) and downstream effectors such as cAMP-dependent protein kinase A (PKA), phospholipases, mitogen-activated protein kinases and ion channels (Yu, 2006). A number of G*α*_i_ subunit genes of filamentous fungi have been characterized (Choi *et al.*, 1995; Gronover *et al.*, 2001; Horwitz *et al.*, 1999; Jain *et al.*, 2002; Turner & Borkovich, 1993; Yu *et al.*, 1996), with *gna1* in *Neurospora crassa* (Turner & Borkovich, 1993) and *fadA* in *Aspergillus nidulans* (Yu *et al.*, 1996) amongst the first to be described. In plant-pathogenic fungi, such as *Magnaporthe grisea*, *Cryphonectria parasitica*, *Colletotrichum trifolii* and *Botrytis cinerea*, some G*α* subunits positively regulate signalling mechanisms important for fungal vegetative growth and pathogenesis (Gao & Nuss, 1996; Gronover *et al.*, 2001; Liu & Dean, 1997; Truesdell *et al.*, 2000). In the chestnut blight pathogen *C*. *parasitica*, deletion of *CPG-1* results in reduced growth and pigmentation, female infertility, loss of virulence and asexual sporulation, and altered gene expression, which are highly similar to the phenotypic changes caused by hypovirus infection (Dawe & Nuss, 2001). Deletion of *CPG-2*, the gene encoding a second G*α* subunit in *C*. *parasitica*, causes only a slight reduction in growth rate and asexual development, and has no effect on either pathogenicity or hypovirulence-related phenotypes (Dawe & Nuss, 2001), suggesting that these G*α* subunits have different functions in *C. parasitica*. In the rice blast fungus *M*. *grisea*, three G*α* subunit genes have been identified and characterized (Liu & Dean, 1997). Deletion of *MAGB*, the gene orthologous to *C*. *parasitica CPG-1*, *A*. *nidulans fadA* and *N*. *crassa gna1*, results in significantly reduced hyphal growth, sporulation, appressorium formation and pathogenicity, whereas disruptions of the other G*α* genes, *MAGA* and *MAGC*, do not affect growth or appressorium formation.

While substantial basic knowledge regarding the functional role of heterotrimeric G protein signalling complexes has been obtained from other fungi, such as *A*. *nidulans* and *N. crassa*, our understanding of the biological significance of these genes in *Gibberella* species (anamorph: *Fusarium* species) is very limited. However, given the very different biological life styles found in *Gibberella* species, e.g. host range, sexual development and virulence mechanisms, we can anticipate greater functional diversity for members of the G protein complex. For instance, G*β* subunits in *Fusarium oxysporum* and *Gibberella moniliformis* differ only in one amino acid, even though their functional roles in plant pathogenesis are quite different. Jain *et al.* (2003) showed that *F. oxysporum FGB1*, a gene encoding a G*β* subunit, is involved in virulence, while *G. moniliformis GBB1* has no impact on maize stalk rot pathogenesis (Sagaram & Shim, 2007). The availability of three *Fusarium* genomes enables comparative functional analysis of heterotrimeric G protein subunits in *G*. *zeae, G. moniliformis* and *F. oxysporum*.

Our working hypothesis is that proper functioning of the G protein complex is required for sexual reproduction, mycotoxin production and scab virulence in *G. zeae*. We tested this hypothesis by deleting the *G. zeae* genes that encode heterotrimeric G protein G*α* and G*β* subunits, and evaluating strains carrying the deletion mutations. We also compared structural and functional properties of the G*β* subunits from *G. zeae, G. moniliformis* and *F. oxysporum* in order to better understand the divergence of gene function in the three species.

## METHODS

### Fungal strains, media and culture.

*G. zeae* strain GZ3639 [obtained from Robert L. Bowden of the Plant Science and Entomology Research Unit, United States Department of Agriculture–Agricultural Research Service (USDA–ARS), Manhattan, KS, USA] was used as the wild-type strain. *G. zeae* Δ*MAT1-2* deletion strain T39ΔM2-1 (Lee *et al.*, 2003), derived from GZ3639, was used as the tester strain for outcrosses. All strains used in this study were stored in 25 % (v/v) glycerol at −80 °C and reactivated on potato dextrose agar (PDA; Difco Laboratories). Carrot agar plates (Leslie & Summerell, 2006) were used for induction of sexual development. For genomic DNA isolation, fungal strains were grown in 50 ml liquid complete medium (CM; Leslie & Summerell, 2006) at 25 °C, 150 r.p.m. for 3 days, and subsequently the hyphal mass was harvested and lyophilized. To produce macrospores, some mycelial agar blocks were inoculated into CMC liquid medium (15 g carboxymethyl cellulose l^−1^, 1 g yeast extract l^−1^, 0.5 g MgSO_4_ l^−1^, 1 g NH_4_NO_3_ l^−1^, and 1 g KH_2_PO_4_ l^−1^ in distilled water) and incubated at 25 °C, 150 r.p.m. for 4–5 days. Macroconidia were collected by filtering through several layers of sterile cheese cloth and were used as an inoculum source. For total RNA extraction, wild-type and mutant strains were grown in 25 ml liquid starch–glutamate (SG) medium (Bacon *et al.*, 1977). For ZEA production and total RNA extraction, all strains were grown on SG medium for 15 days at 25 °C with brief shaking twice a day for the first 3 days. Rice grains (30 g) were used as the solid medium for DON and ZEA production. Wild-type and mutant strains were inoculated and incubated for 3 weeks at 25 °C in the dark, as described previously (Lee *et al.*, 2002).

### Nucleic acid manipulations, PCR primers and sequence analysis.

Fungal genomic DNA and total RNA were extracted as previously described (Han *et al.*, 2004). Standard procedures were used for restriction endonuclease digestion, agarose gel electrophoresis and gel blot hybridization (Sambrook & Russell, 2001). PCRs were performed as previously described (Lee *et al.*, 2002). Nucleotide sequences were assembled and analysed using the dnastar software package. Sequences were compared with the *Fusarium* group genome databases (http://www.broad.mit.edu/annotation/genome/fusarium_group/MultiHome.html) by using the blast algorithm (National Center for Biotechnology Information; http://www.ncbi.nlm.nih.gov).

### Double-joint PCR.

For targeted gene deletion, a gene disruption construct carrying a geneticin-resistance gene (*gen*) flanked by DNA sequences homologous to the sequences located at the 5′ and 3′ ends of the genomic target region was amplified by using a previously described double-joint PCR method with slight modifications (Yu *et al.*, 2004). Briefly, DNA fragments corresponding to regions 5′ and 3′ of the target ORF were amplified from wild-type genomic DNA with specific primer pairs (Table 1). The 5′ (982 bp) and 3′ (992 bp) flanking regions of *GzGPA1* were amplified with primer pairs GPA1-5F and GPA1-5R and GPA1-3F and GPA1-3R, respectively. The 5′ (974 bp) and 3′ (958 bp) flanking regions of *GzGPA2* were amplified with GPA2-5F and GPA2-5R and GPA2-3F and GPA2-3R, respectively. The 5′ (953 bp) and 3′ (920 bp) flanking regions of *GzGPA3* were amplified with GPA3-5F and GPA3-5R and GPA3-3F and GPA3-3R, respectively. The 5′ (928 bp) and 3′ (928 bp) flanking regions of *GzGPB1* were amplified with primer pairs GPB1-5F and GPB1-5R and GPB1-3F and GPB1-3R, respectively. A 1.9 kb DNA fragment containing *gen* was amplified from the vector pII99 (Kim *et al.*, 2005) with primer pair Gen-F and Gen-R. The reverse primers for amplifying the 5′ region and the forward primers for the 3′ region contained ∼20 bp tail sequences that overlapped with the 5′ and 3′ end of the *gen* cassette (Table 1). Three amplicons (the 5′ flanking region, the *gen* cassette and the 3′ flanking region) were mixed at 1 : 2 : 1 molar ratio and used as the template for a second round of PCR with new primer pairs, which were nested in the primers used for the first round of PCR (GPA1-5N and GPA1-3N, GPA2-5N and GPA2-3N, GPA3-5N and GPA3-3N, and GPB1-5N and GPB1-3N). Following purification, PCR products were mixed with fungal protoplasts for transformation, as previously described (Kim *et al.*, 2007).

### Fungal transformation.

Protoplasts of the wild-type strain were prepared by treatment of fresh mycelia grown on YPG liquid medium (3 g yeast extract l^−1^, 10 g peptone l^−1^, 20 g glucose l^−1^ in distilled water) for 12 h at 25 °C with 1 M NH_4_Cl containing 1 % Driselase (1 mg ml^−1^ in NH_4_Cl; InterSpex Products), as described previously (Lee *et al.*, 2002). Approximately 5 μg of fusion PCR product obtained by double-joint PCR was directly added along with 1.2 ml 60 % (w/v) PEG (MW 3350) to protoplast suspensions. Fungal transformants expressing *gen* were selected on regeneration medium containing 75 p.p.m. geneticin. For complementation analyses, the intact copies of four genes amplified from genomic DNA of the wild-type strain were directly added into fungal protoplasts along with the vector pIGPAPA (Horwitz *et al.*, 1999) carrying the hygromycin-resistance gene (*hygB*) as a selective marker.

### Pathogenicity and sexual cross test.

Macroconidia were collected from cultures grown on carrot agar plates for 2 weeks at 25 °C and were suspended in sterile water at a concentration of 1×10^6^ conidia ml^−1^. This conidial suspension was sprayed onto heads of barley at early anthesis. For each treatment, 10 replicate heads were sprayed. The plants were incubated for 2 days in a growth chamber at 25 °C with 100 % relative humidity and then were transferred to a greenhouse. Both selfing and outcrossing of the *G. zeae* strains were performed as previously described (Han *et al.*, 2004).

### Mycelial staining.

Young mycelia of *G. zeae* strains grown in YPG medium were harvested by centrifugation at 12 000 ***g*** for 5 min, washed twice with distilled water and resuspended in distilled water. A 20 μl volume of the mycelial suspension was placed on a glass slide and 2 μl Calcofluor white stock solution (10 mg ml^−1^) was added to it. After incubation for 15 min at 4 °C, the mycelia were observed under an LSM5-Duo (Carl Zeiss) laser scanning microscope.

### ZEA and DON analyses.

To detect ZEA in liquid culture, the fungal culture was filtered through Whatman number 2 filter paper. The filtrate (20 ml) was defatted with 20 ml n-hexane and extracted twice with 20 ml ethyl acetate. The ethyl acetate layer was collected, dried and dissolved in 2 ml methanol. A Shimadzu LC-10 AD HPLC equipped with an RF-10A fluorescence detector (Shimadzu) was used for ZEA analysis. The column was a Symmetry C_18_ column (15 mm × 4.6 mm; particle size, 5 μm; Waters). The detection wavelength was from 274 to 466 nm. The mobile phase was 65 % aqueous methanol at a flow rate of 1 ml min^−1^.

To quantify ZEA and DON production on rice substrate, rice cultures were harvested and extracted with a method from a previously described protocol (Lee *et al.*, 2002). A portion of each extract was injected into the HPLC to analyse ZEA, as described above. Each chemical analysis of toxins was replicated three times.

For DON analysis, 500 μl extract was concentrated to dryness and dissolved in 50 μl trimethylsilylating (TMS) reagent [BSA/trimethylchlorosilane (TMCS)/TMS1, 3 : 2 : 3, Supelco], mixed gently and heated at 60 °C for 5 min. After the reaction, 200 μl n-hexane was added to the solution, which was vortexed with 20 μl distilled water and allowed to stand until the two layers separated. The upper layer (1 μl) was analysed with a Hewlett Packard 5890 series II gas chromatograph with a DB-5 fused silica capillary column [0.25 mm (inside diameter)× 30 mm; 0.25 μm film] (J&W Scientific). The column temperature was maintained at 120 °C for 5 min and then increased to 275 °C at 8 °C min^−1^. The injector and detector temperatures were 280 and 300 °C, respectively.

## RESULTS

### Identification of heterotrimeric G proteins in *G. zeae*

The major heterotrimeric G proteins and downstream effectors are all present in the *G. zeae* genome. We selected components of G protein signalling in *A. nidulans*, i.e. FadA (AAC49476), GanB (AAF12813), GanA (AAD34893), SfaD (AAC33436), GpgA (ABG73391), PkaA (Ni *et al.*, 2005), PkaB (Ni *et al.*, 2005) and PkaR (O59922) (Chang *et al.*, 2004; Rosén *et al.*, 1999; Seo *et al.*, 2005; Yu *et al.*, 1996), and performed a comparison against the *F. graminearum* genome (http://www.broad.mit.edu/annotation/genome/fusarium_group/MultiHome.html) with the tblastn algorithm. We identified *G. zeae* genes corresponding to the major heterotrimeric G proteins and downstream effectors, which we designated *GzGPA1*, *GzGPA2*, *GzGPA3*, *GzGPB1*, *GzGPG1*, *GzPKA1*, *GzPKA2* and *GzPKAR* (Table 2).

*GzGPA1* (FG05535.1, annotated as a conserved hypothetical protein in the *F. graminearum* genome database) encodes a 353 aa protein with high similarity (93 % identity) to *A. nidulans* FadA. GzGPA1, like the other members of the G*α*_i_ family, contains conserved myristylation (Buss *et al.*, 1987, MGXXXS) and pertussis toxin-labelling sites (West *et al.*, 1985, CXXX) in the N- and C-terminal regions, respectively. GzGPA1 and FadA can be grouped in the G*α*_i_ family or subgroup I, which inhibit adenylyl cyclase activity. The *GzGPA2* gene (FG09614.1, annotated as G protein alpha-3 subunit in the genome database), located on contig 1.398 with five introns, encodes a protein with high similarities to *A. nidulans* GanB, *M. grisea* MagA and *C. parasitica* Cpg-2, which are classified as the G*α*_s_ family or subgroup III (Bölker, 1998). *GzGPA3* (FG09988.1, annotated as G protein alpha-2 subunit in the genome database), located on contig 1.415, is interrupted by four introns, and encodes a 354 aa protein similar to GanA of *A. nidulans*, MagC of *M. grisea* and Cpg-3 of *C. parasitica*, which are classified as subgroup II (Bölker, 1998). GzGPA2 contains an *N*-myristylation site at the N-terminus, but GzGPA3 has neither *N*-myristylation nor pertussis toxin-labelling sites. All three G*α* subunits of *G. zeae* contain a conserved GTPase domain.

A tblastn search with *A*. *nidulans* SfaD and GpgA led to the identification of the G*β* subunit gene, *GzGPB*, and G*γ* subunit gene, *GzGPG1*, in the *F. graminearum* genome. *GzGPB1* (FG04104.1, annotated as G protein subunit beta in the genome database) encodes a 359 aa protein that contains six conserved WD40 domains, whose proposed biochemical function is to coordinate multi-protein assemblies and protein–protein interactions that lead to signal transduction and transcriptional regulation (Lengeler *et al.*, 2000).

### Targeted deletion of G*α* and G*β* orthologues in *G. zeae*

**Fig. 1. f1:**
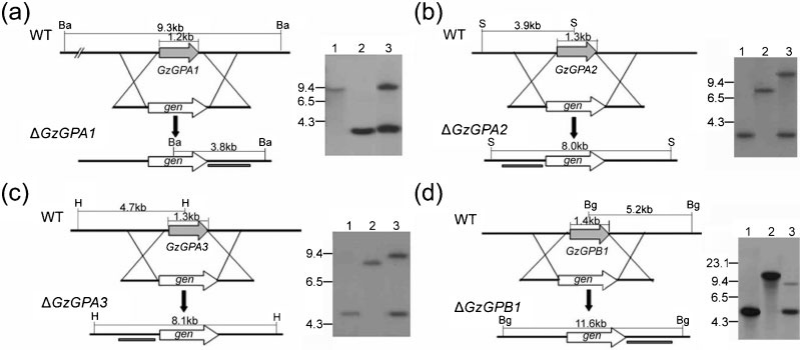
Targeted deletion of *GzGPA1* (a), *GzGPA2* (b), *GzGPA3* (c) and *GzGPB1* (d) from the genome of *G. zeae* wild-type strain GZ3639 (WT). Left of each panel: schematic representation of homologous gene recombination strategy resulting in gene-deletion strains. Ba, *Bam*HI; S, *Sal*I; H, *Hin*dIII; Bg, *Bgl*II restriction sites; *gen*, geneticin-resistance gene. The transcription direction of each gene is indicated by an arrow. Right of each panel: Southern blot analysis of WT (lane 1), a deletion strain (lane 2), and a transformant carrying the disruption construct at an ectopic site (lane 3). Genomic DNAs were digested with the restriction enzymes whose recognition sites are indicated on the left. The blot was hybridized with a ^32^P-labelled DNA fragment, indicated by a solid bar in the deletion scheme. The sizes of standards (in kb) are indicated to the left of each blot.

The deletion of the 1.2 kb *GzGPA1* gene was confirmed by the presence of a single 3.8 kb hybridizing band in a blot of *Bam*HI-digested genomic DNA of the *GzGPA1* deletion (Δ*GzGPA1*) strain, rather than the 9.3 kb band that hybridized to the probe in the wild-type strain (Fig. 1a[Fig f1]). Deletion of the three other orthologous genes was also confirmed by Southern blot analyses. The size differences in the hybridizing bands between the wild-type and the mutant strains, i.e. a single 8.0 kb band in the Δ*GzGPA2* strains instead of a 3.6 kb wild-type band (Fig. 1b[Fig f1]), an 8.1 kb band in the Δ*GzGPA3* strains instead of a 4.7 kb wild-type band (Fig. 1c[Fig f1]), and a 11.6 kb band in the Δ*GzGPB1* strains rather than a 5.2 kb wild-type band (Fig. 1d[Fig f1]), were as predicted and provided molecular evidence of successful homologous gene replacement events.

### *GzGPA2* and *GzGPB1* are required for pathogenicity and normal growth

**Fig. 2. f2:**
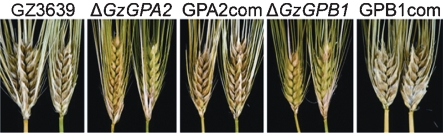
Fungal virulence assay on barley. GZ3639, wild-type strain; Δ*GzGPA2*, *GzGPA2*-deleted strain; GPA2com, *GzGPA2*-complemented strain derived from Δ*GzGPA2*; Δ*GzGPB1*, *GzGPB1*-deleted strain; GPB1com, *GzGPB1*-complemented strain derived from Δ*GzGPB1*.

**Fig. 3. f3:**
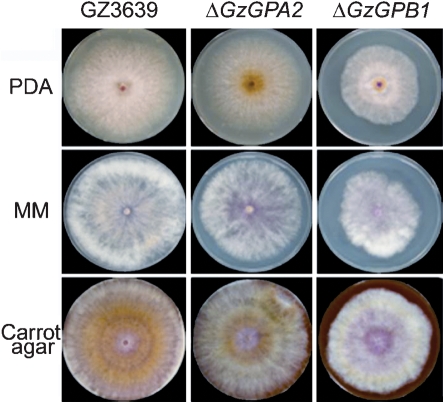
Hyphal growth of GZ3639 (wild-type strain), Δ*GzGPA2* and Δ*GzGPB1* strains on PDA, minimal medium (MM) and carrot agar at 4 days after inoculation.

The Δ*GzGPA1* and Δ*GzGPA3* strains had the same level of pathogenicity towards barley as did the wild-type strain. Δ*GzGPA2* and Δ*GzGPB1* mutants were much less virulent than the wild-type strain (Fig. 2). When intact copies of *GzGPA2* or *GzGPB1* genes were introduced into deletion mutants, their virulence was fully restored, suggesting that both *GzGPA2* and *GzGPB1* are essential for the virulence of *G*. *zeae*. Deletion of *GzGPA1*, *GzGPA2* and *GzGPA3* slightly affected vegetative growth on PDA, with the growth rates of the mutants being similar to that of the wild-type strain, while deletion of *GzGPB1* resulted in 75 % of the hyphal growth of the wild-type strain (Fig. 3).

### *GzGPA1* is required for sexual reproduction

**Fig. 4. f4:**
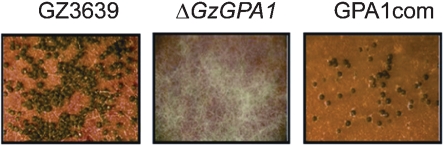
Perithecia formation on carrot agar medium. GZ3639, wild-type strain; Δ*GzGPA1*, *GzGPA1*-deleted strain; GPA1com, *GzGPA1*-complemented strain derived from Δ*GzGPA1.*

We compared sexual reproduction between the wild-type strain and its mutants on carrot agar medium. Deletion mutants of *GzGPA2*, *GzGPA3* and *GzGPB1* produced normal sexual fruiting bodies (perithecia). However, Δ*GzGPA1* mutants failed to develop perithecia (Fig. 4). When we introduced an intact copy of the *GzGPA1* gene back into a Δ*GzGPA1* mutant, sexual reproduction was partially restored (Fig. 4). This result implies that G*α* *GzGPA1* plays an important role in *G*. *zeae* sexual development. The Δ*GzGPA1* mutant did not show significant changes in vegetative growth or asexual sporulation on PDA or carrot agar medium. The pathogenicity of the Δ*GzGPA1* mutants was comparable to that of the wild-type strain on barley.

### *GzGPA2* negatively regulates cell wall chitin content

**Fig. 5. f5:**
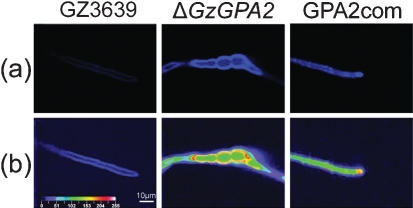
Chitin accumulation in *G. zeae* Δ*GzGPA2* strain. GZ3639, wild-type strain; Δ*GzGPA2*, *GzGPA2*-deleted strain; GPA2com, *GzGPA2*-complemented strain derived from Δ*GzGPA2*. (a) Chitin in the mycelia of each strain was stained with a Calcofluor white solution and observed under UV (420 nm) light. (b) Quantification of fluorescence by laser scanning microscopy. The intensity scale for quantification is indicated on the bottom left, ranging from violet for a weak signal to white for strong fluorescence.

In addition to reduced virulence, the Δ*GzGPA2* mutants exhibited other different phenotypes compared with their wild-type progenitor. We could not produce protoplasts from the Δ*GzGPA2* mutants even when we added five times as much Driselase, a commonly used cell wall-degrading enzyme for *G. zeae,* and incubated for a longer time (up to 15 h). However, when the lysing enzyme solution for *Aspergillus* spp. (Sigma–Aldrich) was added to the Driselase solution, we were able to obtain as many protoplasts from the Δ*GzGPA2* mutants as from the wild-type strain using only 1 % Driselase (data not shown). The cell wall chitin level in the Δ*GzGPA2* mutant, as measured by the intensity of emitted fluorescence from Calcofluor white-stained mycelia, was much higher than that of the wild-type strain (Fig. 5). These results strongly indicate that the *GzGPA2* deletion led to higher chitin accumulation in the Δ*GzGPA2* cells. Moreover, the outcross of the Δ*GzGPA2* mutant to the *MAT1-2* deletion strain (T39ΔM2-1) produced no perithecia, although the self cross of the Δ*GzGPA2* mutant formed as many normal perithecia as that of the wild-type strain (data not shown). All of these altered phenotypes were recovered in transgenic *GzGPA2* mutants expressing an intact *GzGPA2* copy but the chitin content in the complemented cells was still higher than that in the wild-type strain (Fig. 5).

### G*α* *GzGPA1* and G*β* *GzGPB1* negatively regulate toxin production

**Fig. 6. f6:**
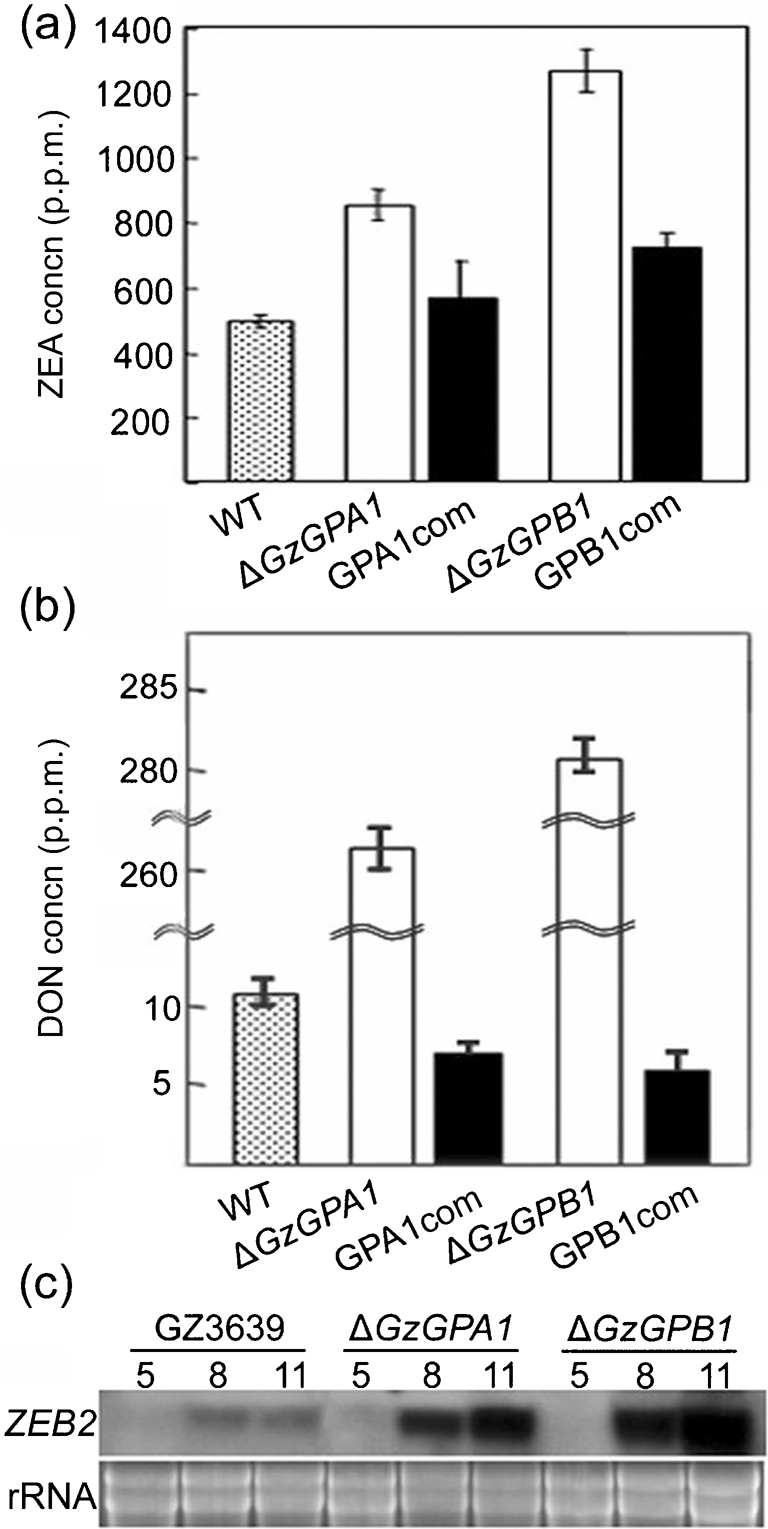
Mycotoxin production in the wild-type (WT), Δ*GzGPA1* and Δ*GzGPB1* strains. Quantification of ZEA (a) and DON (b) produced by the Δ*GzGPA1,* Δ*GzGPB1* and complemented strains on rice medium. (c) Northern blot analysis of wild-type (GZ3639) and mutant strains probed with *ZEB2*. Total RNA samples were extracted from WT, Δ*GzGPA1* and Δ*GzGPB1* grown on SG medium. The probe and the incubation time (days) are indicated at the left and above the blot, respectively. The ethidium bromide-stained rRNAs used as a loading control are indicated.

Δ*GzGPA1* and Δ*GzGPB1* mutants produced significantly more DON and ZEA than did the wild-type strain. The wild-type strain produced 460 p.p.m. ZEA; Δ*GzGPA1* and Δ*GzGPB1* mutants produced 800 and 1180 p.p.m., which was ∼60 and ∼250 % higher, respectively, than the wild-type strain (Fig. 6a[Fig f6]). Moreover the Δ*GzGPB1*strain produced 50 % more ZEA than the Δ*GzGPA1* strain. The wild-type strain produced about 10 p.p.m. DON, whereas Δ*GzGPA1* and Δ*GzGPB1* mutants produced 270 and 280 p.p.m., respectively (Fig. 6b[Fig f6]). Consistent with increased ZEA production, the mRNA level of *ZEB2,* a gene encoding the transcription factor involved in ZEA biosynthesis, was upregulated in Δ*GzGPA1* and Δ*GzGPB1* mutants relative to the wild-type strain (Fig. 6c[Fig f6]). When we introduced an intact copy of each gene back into the deletion mutants, the level of toxin production returned to approximately that of the wild-type strain, confirming that deletion of the genes was responsible for enhanced toxin production (Fig. 6a[Fig f6], b).

## DISCUSSION

Heterotrimeric G proteins are highly conserved in model filamentous and plant-pathogenic fungi. The key units of the G protein signalling complex are G protein-coupled receptors, G proteins (consisting of G*α*, G*β* and G*γ* subunits) and downstream effectors. We identified and characterized three putative G*α* subunits and one G*β* subunit from the *F. graminearum* genome in this study. These G protein subunits are essential for fungal development, secondary metabolism and virulence (Bölker, 1998; Lengeler *et al.*, 2000). One of the most extensively studied G protein signalling models in filamentous fungi is *A. nidulans*, in which G protein subunits control asexual/sexual development, vegetative growth and sterigmatocystin (ST) biosynthesis (Yu & Keller, 2005).

In *Gibberella* (*Fusarium*) species, however, the picture is far from clear. Two G*α* subunits from *F. oxysporum*, *FGA1* and *FGA2*, and the G*β* subunit from *F. oxysporum*, *FGB1*, and *G. moniliformis*, *GBB1*, have been functionally characterized (Delgado-Jarana *et al.*, 2005; Jain *et al.*, 2002, 2003, 2005; Sagaram & Shim, 2007). However, they do not have direct parallels in *Gibberella* (*Fusarium*) species. Given the number of phenotypic variations known in *Gibberella* (*Fusarium*) species, e.g. in host range, reproductive strategy and secondary metabolite production, there is a need to unambiguously characterize the functional role of the G protein components in *G. zeae*.

Heterotrimeric G protein signalling controls sexual reproduction in model and plant-pathogenic fungi (Liu & Dean, 1997; Seo *et al.*, 2005). In *G*. *zeae*, the G*α* and G*β* subunits seem to regulate either sexual reproduction or pathogenic processes, but not both. Deletion of *GzGPA1* results in female sterility, whereas deletion of *GzGPB1* does not alter sexual fertility. Our results suggest that *GzGPA1* alone controls sexual reproduction in *G. zeae*. Similar results have been observed in *G. moniliformis* (Sagaram & Shim, 2007). Considering the genetic relatedness of *G. zeae* and *G. moniliformis*, we hypothesize that the *GzGPA1* orthologue (FVEG_06962.3) in *G. moniliformis*, which is 100 % identical at the amino acid level, serves as the primary, if not exclusive, regulator of sexual mating in *G. moniliformis.*

The *G. zeae* G protein subunits also have an important and complicated role in plant pathogenesis. Deletion of *GzGPA2* or *GzGPB1* significantly reduced fungal virulence toward host plants. Pathogenicity tests were done with 1×10^6^ conidia ml^−1^ on barley. Lower concentrations should be tested to exclude the possibility that *GzGPA1* and *GzGPA3* mutants show a more subtle phenotype. The reduced virulence in the Δ*GzGPA2* mutant may be attributed to its higher level of chitin, which may elicit plant defence mechanisms. This is plausible, since chitin has been shown to be, or implicated as, a signal in plant defence (Barber *et al.*, 1989; Tsutsui *et al.*, 2006; Wan *et al.*, 2004). The fungal G proteins are known to regulate cell wall composition in several cases, but their roles may not be directly comparable to those in *G. zeae*. In *A. nidulans, FadA* and *SfaD* (orthologous to *GzGPA1* and *GzGPB1*, respectively) seem to negatively regulate cell wall chitin content (Coca *et al.*, 2000), while only Δ*GzGPA2* is responsible for the hyperaccumulation of chitin in *G. zeae*. The deletion of *Trichoderma atroviride tga3*, orthologous to *GzGPA2*, results in resistance to protoplasting by cell wall-lysing enzymes, as did Δ*GzGPA2,* but no changes in cell wall chitin content (Zeilinger *et al.*, 2005). Final confirmation of this hypothesis, however, awaits further evidence supporting the hypothesis that the chitin-overproducing Δ*GzGPA2* mutant elicits plant defence responses, and that *GzGPA2* directly controls some chitin synthesis-related genes.

The role of fungal G*β* subunits in plant pathogenesis is not conserved. Deletion of the *M*. *grisea* G*β* subunit, *mgb1,* and *Cochliobolus heterostrophus CGB1* leads to defective appressorium formation that prevents host penetration and infection (Ganem *et al.*, 2004; Nishimura *et al.*, 2003). Disruption of the *C*. *parasitica* and *F*. *oxysporum* G*β* subunit genes also results in significantly reduced virulence (Jain *et al.*, 2003; Kasahara & Nuss, 1997). However, in *Ustilago maydis*, deletion of the G*β* subunit gene, *BPP1,* does not block tumour formation, although a slight reduction in virulence does occur (Muller *et al.*, 2004). Deletion of the G*β* subunit gene in *G. moniliformis* does not alter the ability of the strain to infect and colonize maize stalk tissue (Sagaram & Shim, 2007). Similar ranges of chitin accumulation in the Δ*GzGPB1* mutant compared to the wild-type strain indicate that the G*β* protein may control fungal virulence in a different manner to the G*α* GzGPA2 protein in *G. zeae*.

In *A. nidulans*, both activated G*α* (FadA) and G*βγ* (SfadA : GpgA) propagate the vegetative growth signal through the cAMP–PKA signalling pathway (Rosén *et al.*, 1999; Seo *et al.*, 2005; Shimizu & Keller, 2001; Yu *et al.*, 1996). However, independent gene deletion of the three G*α* subunits slightly affects hyphal growth (∼10 % reduction), implying that G*α* proteins have minor roles in hyphal growth of *G. zeae*.

Another important role of the heterotrimeric G protein signalling complex in filamentous fungi is to regulate secondary metabolite production. This complicated biosynthetic process is closely associated with physiological and morphological development (Calvo *et al.*, 2002). FadA-mediated vegetative growth signalling represses ST biosynthesis in *A. nidulans* and asexual and sexual development, but enhances penicillin production (Yu *et al.*, 1996). Expression of the *A. nidulans fadA^G42R^* dominant activating mutant allele in *Fusarium sporotrichioides* increases production of T-2 toxin (Tag *et al.*, 2000), but inhibits production of aflatoxin in *Aspergillus* *parasiticus* (Hicks *et al.*, 1997) and *Aspergillus* *flavus* (McDonald *et al.*, 2005). Deletion of the G*β* subunit gene *sfaD* of *A*. *nidulans* drastically reduces ST biosynthesis. In *G. zeae*, deletion of either *GzGPA1* or *GzGPB1* results in increased mycotoxin production, suggesting that both G protein subunits have a role in the negative regulation of mycotoxin production. The significant upregulation of *ZEB2* observed in the Δ*GzGPA1* and Δ*GzGPB1* mutants is consistent with this conclusion (Fig. 6c[Fig f6]). In *G. moniliformis*, however, disruption of the G*β* subunit results in significant down-regulation of *FUM1*, a polyketide synthase gene essential for fumonisin biosynthesis, and severely curtails fumonisin production (Sagaram & Shim, 2007). The role of G*α* subunits of *G. moniliformis* in the regulation of fumonisin biosynthesis remains unknown.

Even at the DNA level, the G*β* gene, including its location and the number of introns, is highly conserved amongst *Gibberella* species (data not shown). When analysed at the amino acid level, there is only a single amino acid difference between *F. oxysporum* (FOXG_11532.2) and *G. zeae* (FGSG_04104.3) or *G. moniliformis* (FVEG_10291.3). The G*β* proteins from the *Gibberella* species all contain six WD40 repeats, whereas the homologous proteins from *A. nidulans* and *N. crassa* contain seven and four WD40 repeats, respectively. When compared to previous studies of *G. moniliformis* and *F. oxysporum*, the G*β* protein from *G. zeae* regulates fungal development, virulence and secondary metabolism in a manner different from that in the other two species. We hypothesize that certain biological traits, e.g. host adaptation and/or sexual life style, may have altered gene function during evolution, but the basis for this functional divergence by such highly conserved proteins remains to be tested and explained.

The orthologues of the G*γ* subunit GzGPG1, the protein kinase catalytic subunits GzPKA1 and GzPKA2, and the regulatory subunit GzPKAR are all present in *G. zeae*. These genes are the components of a cAMP-dependent PKA signalling pathway, one of the major downstream regulatory cascades in heterotrimeric G protein-mediated regulatory mechanisms in fungi (Shimizu & Keller, 2001). Identification of these components in the *G. zeae* genome suggests that the G protein–cAMP–PKA signalling pathway is involved in controlling *G. zeae* growth, development, pathogenicity and toxin production. Further functional characterization of the G protein complex and the downstream signalling pathways is needed to understand the molecular mechanisms of virulence and mycotoxin production and their regulation in *G. zeae*.

## Figures and Tables

**Table 1. t1:** Primers used in this study

**Primer**	Sequence* (5′→3′)	**Base-pair positions† at contig**
GPA1-5F	CATCTCGGATCTGCCTCTGATT	1439–1460 at 1.228
GPA1-5R	GCACAGGTACACTTGTTTAGAGAGTGACGGTAGTTTGGCTTTGCT	2420–2399 at 1.228
GPA1-3F	CCTTCAATATCACTTCTGTCGAAATGATTTTTTTCTTG	3691–3706 at 1.228
GPA1-3R	GTCTATCGACAGGTCACCGTGT	4683–4662 at 1.228
GPA1-5N	ATTTCTCACTCCCAGCTC	1462–1479 at 1.228
GPA1-3N	GCATGTCTCATCACCAGA	4622–4605 at 1.228
GPA2-5F	GAACGGTCGAGGTCTCTTTGTA	5674–5653 at 1.398
GPA2-5R	GCACAGGTACACTTGTTTAGAGATTGAGAATTAGAAA AA AGCGGC	4700–4721 at 1.398
GPA2-3F	CCTTCAATATCATCTTCTGTCGATAGTGGAACGCTGCTTTTTTCT	3344–3323 at 1.398
GPA2-3R	GCCAACAAGCCATATACCGATA	2386–2407 at 1.398
GPA2-5N	CGCTCCAATTAGTCCATC	5611–5594 at 1.398
GPA2-3N	CACATCATTCGTTCCCAA	2440–2457 at 1.398
GPA3-5F	CCTAGAGAGGTACGCTCCAAAG	55614–55635 at 1.415
GPA3-5R	GCACAGGTACACTTGTTTAGAGAATTGTGTGTTGTTGAAGCGACT	56564–56543 at 1.415
GPA3-3F	CCTTCAATATCATCTTCTGTCGAGATATACGGCGTTTGGACATTT	57978–57999 at 1.415
GPA3-3R	CTTTTGCAGTTTCGTTGTGTGT	58898–58877 at 1.415
GPA3-5N	CCACTGCCAAAAAAACCT	55644–55661 at 1.415
GPA3-3N	GAGCATCAAGCAGACCAG	58872–58855 at 1.415
GPB1-5F	TTTTTGCAGATGCGAAATAGGT	66401–66422 at 1.179
GPB1-5R	GCACAGGTACACTTGTTTAGAGAGCGACAGGAGAGATTAAACGAG	67328–67307 at 1.179
GPB1-3F	CCTTCAATATCATCTTCTGTCGACGTTCACGACCATGAAACTATT	68794–68815 at 1.179
GPB1-3R	TCATCAATGGGATATGCTAAGC	69722–69701 at 1.179
GPB1-5N	GTACATCTAGGCCAGCCA	66469–66486 at 1.179
GPB1-3N	AAAAGACCGAGCAACAGA	69688–69671 at 1.179
Gen-F	CGACAGAAGATGATATTGAAGG	For amplification of *gen* cassette from pII99 vector
Gen-R	CTCTAAACAAGTGTACCTGTGC	

*The underlined sequences are matched with the primer sequences of either Gen-F or Gen-R. **†**Based on the *F. graminearum* genome database.

**Table 2. t2:** Genes encoding putative G protein signalling components in *G. zeae*

Gene (linkage group)*	**Protein used for tblastn/protein (*A. nidulans*)†**	**Pairwise blastp‡**		**Gene coding and physical information**	
		**E-value**	**Contig**	**ORF region in contig defined in this study/coding strand**	**Locus number**
GzGPA1 (III)	FadA/G*α* subunit	0.0	1.228	2422–3691/+	FG05535.1
GzGPA2 (VI)	GanB/G*α* subunit	1e−153	1.398	4698–3345/−	FG09614.1
GzGPA3 (VII)	GanA/G*α* subunit	2e−98	1.415	56 563–57 911/−	FG09988.1
GzGPB1 (II)	SfaD/G*β* subunit	8e−144	1.179	67 367–68 789/+	FG04104.1
GzGPG1 (IV)	GpgA/G*γ* subunit	1e−25	1.303	158 138–157 801/−	FG07235.1
GzACY1 (I)	CyaA/adenylyl cyclase	0.0	1.62	29 627–22 382/−	FG01234.1
GzPKA1 (IV)	PkaA/cAMP-dependent protein kinase	2e−146	1.303	212 185–214 114/+	FG07251.1
GzPKA2 (V)	PkaB/cAMP-dependent protein kinase catalytic subunit	2e−110	1.353	83 645–84 932/+	FG08729.1
GzPKAR (VII)	PkaR/cAMP-dependent protein kinase regulatory subunit	9e−123	1.411	91 620–90 371/−	FG09908.1

*Linkage groups are determined based on the contig and supercontig alignment with the genetic map and supplied by the website (http://www.broad.mit.edu/annotation/genome/fusarium_group/MultiHome.html). †tblastn between the proteins in *A. nidulans* and *F. graminearum* genome databases was performed. ‡Results of the pairwise blastp analysis between putative *G. zeae* proteins and orthologous *A. nidulans* proteins.

## Abbreviations

- DON, deoxynivalenol
- PKA, protein kinase A
- ST, sterigmatocystin
- ZEA, zearalenone
